# Prediction of driver variants in the cancer genome via machine learning methodologies

**DOI:** 10.1093/bib/bbaa250

**Published:** 2020-10-22

**Authors:** Mark F Rogers, Tom R Gaunt, Colin Campbell

**Affiliations:** Fort Collins, Colorado; MRC Integrative Epidemiology Unit, University of Bristol; University of Bristol with interests in machine learning and medical bioinformatics

**Keywords:** variant prediction, cancer, machine learning

## Abstract

Sequencing technologies have led to the identification of many variants in the human genome which could act as disease-drivers. As a consequence, a variety of bioinformatics tools have been proposed for predicting which variants may drive disease, and which may be causatively neutral. After briefly reviewing generic tools, we focus on a subset of these methods specifically geared toward predicting which variants in the human cancer genome may act as enablers of unregulated cell proliferation. We consider the resultant view of the cancer genome indicated by these predictors and discuss ways in which these types of prediction tools may be progressed by further research.

## Background

Next generation sequencing technologies have led to the identification of many variants in the human genome which could drive disease. To distinguish sequence variants which are causatively neutral from active disease-drivers, a variety of prediction tools have been proposed covering single nucleotide variants (SNVs), short insertions and deletions (indels) and other types of driver. A number of types of data are prospectively informative for distinguishing disease-drivers from neutral variants. For example, one type of data would be sequence conservation: if a variant occurs in a genomic region which is highly conserved across species, then the variant has a higher probability of being pathogenic relative to a variant in a region where there has been substantial variation. Sequence conservation is an indicator of functional significance. Examples of suitable conservation measures would include *PhyloP* [1], *PhastCons* [2] and *FATHMM* [3] scores. Another informative feature would be the normalized ratio of non-synonymous to synonymous variants (dN/dS): a coding variant which does not result in an amino acid substitution has a higher probability of being benign. The functional consequence of a variant is also informative: it may be a missense variant or create a stop codon, for example. Sequence uniqueness within the genome is also useable data: unique regions may have a higher expectation of being functionally significant. These, and a variety of other types of data, may carry information indicating if a variant in the genome could be pathogenic, or neutral in effect, though *a priori* we do not know if a particular type of data is actually useful and to what extent.

In consequence, tools for predicting the possible pathogenic impact of a variant are commonly based on machine learning and use of data integration methodologies over differing types of data. Henceforth, we will refer to these different types of input data as *feature groups*. For example, our own *FATHMM-XF* classifier [4] is used for predicting the disease-driver status of SNVs. If restricting to coding SNVs in the genome, *FATHMM-XF* uses six feature groups which it draws from 27 possible feature groups, which might be informative.

In this context, two broad approaches to data integration could be described as *data-level integration* and the usage of *ensembles of classifiers*. For data-level integration a typical approach may be *multiple kernel learning* (MKL) [5, 6]. Data can appear in a wide variety of forms such as discrete or continuous numbers, sequence strings or graphs, for example. All such prospective feature groups can be encoded into respective *kernel matrices* which encode the similarity of data objects [7]. These component kernel matrices are weighted by the MKL algorithm, according to relative informativeness, and used to construct a composite kernel matrix which is used in the decision function of a classifier. An example of such a variant effect predictor using MKL would be our *FATHMM-MKL* tool [8]. Of course, we do not know if a given feature group is actually informative or the information it contains is implicit in another feature group, and therefore potentially redundant. However, if uninformative or redundant, an efficient MKL algorithm should zero-weight its contribution to the composite kernel. With an ensemble of classifiers, each component classifier can handle an individual data type, and a variably weighted or uniformly weighted combination of these *base* classifiers is used for the classification task. An example of the latter approach would be the *REVEL* predictor [9].

Many further variations on this general approach are possible. We could simply use all the data in one single data matrix: such an approach is used by the predictor *DANN* [10], based on a deep learning neural network. The use of such a single data matrix would allow an algorithm to capture information about co-associations between different types of data. However, this approach could have the disadvantage of potentially incorporating noise from uninformative data, hence degrading performance. This problem can be mitigated by using a greedy *sequential learning method* in which we start with the most informative feature group, with the highest accuracy on validation data, and aggregate further feature groups until the validation accuracy plateaus or falls. At this point we exit the learning process and proceed to test accuracy evaluation on independent unseen data. Such a sequential learning method can also be used with an ensemble of classifiers or applied to multiple kernel learning. In Ying *et al.* [11], we established that sequential learning applied to multiple kernel learning led to a significantly more accurate classifier in some biomedical data integration prediction tasks, and in Rogers *et al.* [12], we established such a test accuracy gain over *FATHMM-MKL* for SNV pathogenicity prediction. The result of using these various integrative approaches is that an accurate classifier can be built based on a set of feature groups which, taken individually, may only be weakly informative.

For diseases which are not cancer, quoted test accuracies of these integrative predictors is high. For example, for the latest update of *CADD* [13], test accuracies of approximately 90% are reported for SNV prediction in coding regions of the human genome. Via benchmarking studies across different methods, such test accuracies have been reproduced, and it is becoming increasingly common to provide a Docker image [14], enabling other groups to replicate or extend findings. Databases have also been published tabulating these predictions and which also afford easier comparisons across different methods [15, 16]. For diseases other than cancer, these tools have been used to identify pathogenic variants driving rare-variant disease, for example, juvenile open angle glaucoma [17], argininosuccinate lyase deficiency [18] and corticobasal degeneration [19]. For these reasons, the American College of Medical Genetics and American College of Pathologists (ACMG/AMP) variant classification guidelines recommend the use of these classification tools [20].

Tables 1 and 2 list some commonly used generic tools in this context. These listings are not exhaustive, and we do not pursue a comparative performance study here, since a number of thorough benchmarking studies have already been published [20, 34, 35]. The methods in Table 1 directly use genomic and other types of data, while those listed in Table 2 use predictions from already published predictors, thereby leveraging a test accuracy improvement by including further types of data. Alternatively, we could simply use an ensemble of previously published predictors, perhaps weighting the contributions of these individual predictors by relative accuracy.

**Table 1:** Some commonly used tools for predicting the pathogenic impact of variants in the human genome. Except for *Eigen* and *Eigen-PC*, most methods use supervised learning. Most methods use data integration, utilizing conservation measures, functional annotations and other feature groups to optimize prediction accuracy

Name	Method and features used	Reference
*CADD*	Logistic regression model trained with a wide variety of genomic features. Uses proxy neutrals estimated from the last human-ape genome divide and simulated *de novo* variants for proxy deleterious.	Kircher et al. [21] Rentzsch et al. [13].
*DANN*	Deep neural network using conservation measures, epigenomics and genomic data.	Quang et al. [10]
*Eigen Eigen-PC*	Unsupervised learning methods using genomics, functional annotations and epigenomics.	Ionita-Laza et al. [22]
*FATHMM-MKL* *FATHMM-XF*	Multiple kernel learning and a later gradient boosting method using conservation measures, genomic and epigenomic features.	Shihab et al. [8] Rogers et al. [4]
*Mutation Taster 2*	Naive Bayes classifier using conservation measures, regulatory and genomic features.	Schwarz et al. [39]
*Polyphen2*	Naive Bayes classifier using sequence and structure-based features.	Adzhubei et al. [24]
*PON-P2*	Random Forest classifier, scoring amino acid substitutions as pathogenic, neutral or unknown, using conservation, functional and structural annotations.	Niroula et al. [25]
*PROVEAN*	Alignment scores based on sequence homology.	Choi et al. [26]
*SIFT* *SIFT4G*	Position-specific scoring matrix derived from sequence homology	Ng et al. [27] Vaser et al. [28]
*VEST*	Random Forest method using conservation measures, protein structural measures, genomic and amino acid features.	Carter et al. [29]

**Table 2:** Some further generic tools for predicting the pathogenic impact of variants in the human genome. These are examples of methods which use pre-existing prediction methods to leverage performance either by using further data or by using an ensemble of pre-existing tools, prospectively weighted by relative accuracy

Name	Method and features used	Reference
DEOGEN2	Random Forest classifier using *PROVEAN* gene pathway, evolutionary and other features.	Raimondi et al. [30]
GAVIN	Using a gene-specific calibration approach enhances test accuracy of *CADD* scores.	Van de Velde et al. [31]
M-CAP	Gradient boosting tree classifier using 9 pre-existing tools (*CADD*, *SIFT*, *FATHMM*, etc) and other features, such as genomic and conservation measures.	Jagadeesh et al. [32]
MutPred2	Random Forest based method using *SIFT*, conservation measures and protein function and structural measures.	Li et al. [33]
REVEL	Random Forest classifier using 13 established tools such as *VEST*, *FATHMM*, *SIFT* and others.	Ioannidis et al. [9]

Some methods in Tables 1 and 2, such as *SIFT*, only cover prediction in coding regions while others, such as *CADD*, cover both coding and noncoding regions. Some methods cover prediction with SNVs, others cover indel prediction only [36], while others, such as *CADD* [13], cover both within the same package. To assist with the interpretation of results from these predictors, genomic visualization tools have been proposed [37], in addition to comprehensive software suites, such as *CRAVAT* [38], which provides extensive annotation, interpretation and visualization.

## Cancer-Specific Predictors

These generic tools have been used with some success for prediction with cancer genome data [39] or used as comparator models with the proposal of more cancer-specific methods [40]. However, benchmarking studies [34] show a clear test accuracy gain for cancer-specific methods over these generic tools. Smaller in number, relative to generic predictors, Table 3 presents some of the main methods. One dedicated tool is *CHASM* [29, 41], which ranks somatic driver variants for specific cancer types using a Random Forest classifier. *CHASM* provides *P*-values and false discovery rate (FDR) measures for deriving the ranking score and it is trained on positives (disease-drivers) from the *COSMIC* cancer archive [48] and simulated neutral variants. For example, Carter *et al.* [49] generated simulated neutral substitutions by random sampling from a multinomial distribution, which has a dependence on the genomic context, and cancer type, and takes into account the dinucleotide double-stranded structure of DNA. *CHASM* is accessible within *CRAVAT* [38], which provides a user-friendly interface for assessing and prioritizing possible driver-genes and driver variants responsible for unregulated cell proliferation. *CRAVAT* handles both germline and somatic variants and can indicate predicted impact on protein function via the associated Variant Effect Scoring Tool (*VEST*) [29]. As discussed below, prediction of the driver status of variants within coding regions of the human cancer genome is currently more tractable than prediction within noncoding regions. *FunSeq2* [45] is specifically focused toward variants within noncoding regions of the cancer genome and uses genomic and cancer annotation data with a variant prioritization pipeline. The pipeline generates a weighted score based on sequence conservation data, loss- or gain-of-function for transcription-factor binding, enhancer-gene linkage and other features. Other cancer-specific methods, such as *CanDrA* and *TransFIC*, use predictions from pre-existing variant effect predictors. For example, *TransFIC* [47] uses a transformation based on variant distribution differences between germline and cancer somatic SNVs to modify scores provided by well-known tools, *SIFT*, *Polyphen2* and *MutationAssessor*, to the case of cancer somatic variants.

**Table 3:** A set of prediction tools specialized to predicting the disease-driver status of variants in the human cancer genome. Although the generic predictors of Tables 1 and 2 have been used successfully for variant prediction in the cancer genome, more specialized methods would be expected to achieve higher test accuracy. As for generic predictors, some methods are trained directly from data, while others, such as CanDrA and TransFIC, use predictions from pre-existing variant effect predictors

Name	Method and features used	Reference
*CHASM*	Random Forest method using evolutionary and structural features.	Carter et al. [29] Tokheim et al. [41]
*CRAVAT4*	An evolving suite of informatics tools for mutation interpretation and impact prediction.	Masica et al. [38]
*CScape*	Gradient boosting (sequential learner) using evolutionary and genomic features	Rogers et al. [42]
*CScape-somatic*	Similar to *CScape* except distinguishes rare from recurrent somatic SNVs using cancer data only.	Rogers et al. [43]
*FATHMM-cancer*	Using evolutionary data, a predecessor to *CScape.*	Shihab et al. [44]
*FunSeq2*	Scoring scheme, using conservation, regulatory and other measures. Prioritizes cancer somatic variants, especially for regulatory noncoding mutations.	Fu et al. [45]
CanDrA	Support Vector Machine method using 10 published predictors (*CHASM*, *SIFT* and others) and evolutionary, structural and gene features.	Mao et al. [73]
TransFIC	Scoring method utlilizing *SIFT*, *Polyphen2* and *MutationAssessor*	Gonzalez-Perez [47]

In a recent paper we proposed *CScape* [42], a machine-learning-based tool for predicting the driver status of SNVs in the human cancer genome. This tool was based on an integrative classifier which could use up to 30 feature groups to predict if an SNV was acting as a driver, or was neutral, with the addition of a confidence measure attached to this class assignment. In Table 4, we describe some of the feature groups used for the construction of *CScape* (see [42] for a more detailed discussion). This table is only illustrative since the feature groups used will depend on the classification task considered, feature groups can be discarded using sequential learning because uninformative but also because the information is implicit in an already used feature group, and feature groups may be inherently useable but discarded, or down-weighted, because data acquisition is sparse across the genome (see our mention of *ENCODE* data in Table 4).

**Table 4:** Some typical feature groups which *may* be informative for discriminating SNV-drivers from neutrals in the context of cancer. Some feature groups may only be informative for coding, or alternatively for noncoding regions: for example, an indicated amino acid substitution under *Consequence* is only relevant to coding regions. During an additive sequential learning process, some feature groups may be discarded because only weakly informative or because the information is implicit in already learnt data. For the *ENCODE* feature group, and for construction of our *CScape* predictor, only four groups of data within this feature group yielded discriminatory information among variants in noncoding regions, and none in coding regions. However, this is not an indicator that this data source is inherently uninformative: in this case sparse coverage of data across the genome appeared to limit its use

Feature group	Description
Conservation	Variants within highly conserved regions are more likely to be disease-drivers relative to variants within regions with high variability across species. Multispecies comparison can be achieved using a variety of evolutionary conservation scores derived, for example, from *Phastcons* [2] or *PhyloP* [1].
Sequence	Sequence comparison of *k*-mers within a region before, and after, a mutation has occurred within a sequence. This type of measure may carry information covering susceptibility of sequence regions to oncogenic mutation.
Genomic context	Covering GC content, repeat regions, measures of region uniqueness and other genomic context measures.
Consequence	Covering the consequences of a variant, such as a resultant amino acid substitution, or the truncation of a transcript. In [42], we used 35 attributes within this feature group covering a wide variety of possible variant consequences such as transcript ablation, a splice acceptor variant, a stop or start loss, or an incomplete terminal codon variant.
ENCODE	Information from the *ENCODE* [50] database, such as the ratio of *non-synonymous* to *synonymous* mutations (*dN/dS*), histone modification data from ChIP-Seq peak calls, open chromatin, methylation, gene expression or transcription factor binding site data from PeakSeq and SPP.

**Figure 1:**
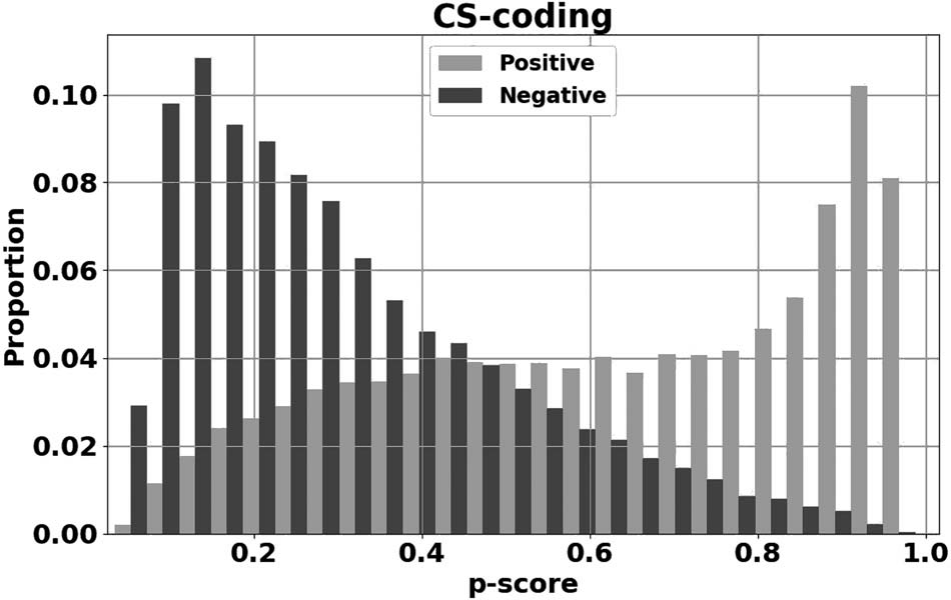
*y*-axis: the proportions for correct prediction of disease-drivers (positives, light gray) and neutrals (negatives, dark gray) against *P*-score (*x*-axis: the confidence an SNV is a driver), evaluated on unseen test data. These predictions are for SNVs in *coding* regions of the human cancer genome and the methodology behind this plot is more fully described in Rogers *et al.* [42].

*CScape* was trained using putative drivers identified as recurrent SNVs observed in tumors and extracted from the *COSMIC* database [48]. The neutrals were represented by variants extracted from the 1000 Genomes database [51]. After training, the method is tested on unseen data to evaluate class assignment on novel instances. One such test evaluation mode is leave-one-chromosome-out cross validation (LOCO-CV), in which the classifier is trained on all the chromosomes except one, with performance evaluated on the held-out chromosome. The held-out chromosome is then consecutively rotated through all chromosomes to gain an averaged test accuracy performance. The method was also evaluated on data from the International Cancer Genome Consortium [52] and The Cancer Genome Atlas [53]. Using LOCO-CV and balanced (50:50) test data, *CScape* had a test accuracy of *72.3%* in coding regions of the cancer genome and *62.3%* in noncoding regions. Performance for coding regions is presented in Figure 1 where the *P*-score on the *x*-axis is the confidence measure for assignment as a driver (*1* is the maximum confidence that the SNV is a predicted driver, *0* the maximum confidence of a neutral SNV). At a threshold of *0.89* on this confidence, the classifier can achieve a test accuracy of *91.7%,* however, this level of accuracy is only achieved at *17.7%* of nucleotide positions in the cancer genome.

For cancer-specific classifiers, such as *CScape*, construction of a set of positives (disease-drivers) looks tractable: for SNVs we could consider variants which are recurrently observed among tumors at a given location and which are also absent from healthy individuals. For the negatives (neutrals), we could create simulated neutral variants or, as with many of the generic tools discussed to date, use germline variants drawn from samples derived from healthy subjects. Databases for the latter could be 1000 Genomes project data [51], or more recent databases such as *GnomAD* [54]. One issue with using germline variants as neutrals is that the somatic variants driving cell proliferation are distributed differently from germline variants. As previously mentioned, sequence conservation is a useful feature group for indicating a pathogenic SNV. However, somatic variants are typically distributed within more evolutionary conserved regions and germline within less conserved. Unfortunately, the sequence conservation feature groups, and other feature groups similarly affected, are typically those groups informative for distinguishing neutrals versus driver SNVs. Discrimination could therefore be influenced by the distinction of germline versus somatic, rather than neutral versus disease-driver.

To investigate this issue, we recently proposed *CScape-somatic* [43], an integrative classifier for predictively discriminating between recurrent and rare variants in the human cancer genome. This predictor is trained purely on cancer genome data. One class consists of rare occurrence somatic variants satisfying *r = 1* (occurs once in the *COSMIC* data used). The other class is significantly recurrent somatic single point mutations in the human cancer genome (highly recurrent variants with a recurrence of *r ≥ 8* in noncoding regions and *r ≥ 7* in coding regions). Of course, rare (*r = 1*) somatic variants could be rare drivers and highly recurrent variants confined to cancer samples could actually be passengers if, say, co-located with a driver. However, it is probable that rare somatic variants are enriched for neutrals and highly recurrent are enriched for drivers. In any case, we find [43] that a classifier can be constructed to predictively distinguish these two classes with reasonable LOCO-CV test accuracy: so they must have distinguishing characteristics. Tested on unseen cancer somatic SNVs, *CScape-somatic* achieves *74%* balanced accuracy for predictive discrimination in coding regions and *69%* in noncoding regions, while even higher accuracy may be achieved using thresholds to isolate high-confidence predictions. This investigation, though, does highlight the importance of finding an unbiased class of negative (neutral) examples, when constructing these predictors. Similarly, there are issues involved in defining the positive set of driver examples, for example, the choice of threshold on the recurrence rate of a variant confined to cancer genomes only.

## The Resultant View of the Cancer Genome

Aside from building prediction tools, it is important to investigate the resultant view of the cancer genome which derives from the use of these tools. This will give insights into the biological validity of these methods, their current shortcomings and opportunities for future development.

In a recent study [55], we explored the view of the cancer genome which derives from *CScape*. This study indicated that the mean number of SNVs acting as drivers in coding regions is very small in size, though very variable by cancer type. Of course, there are a vast number of locations in the genome where drivers can be located. However, a particular clone will typically have a very small set of such SNV-drivers, generally single or low double digits. Hypermutation [56] was excluded from our study. Samples exhibiting hypermutation were easy to identify since the predicted driver count is very distinct, generally of the order of hundreds of predicted SNV-drivers. Our study suggested a mean of *14.9* SNV-drivers in coding regions, taken across the *25* types of cancer included in the survey, and excluding those tumors exhibiting hypermutation (this mean is quoted for an FDR of *5%* [55]). Some types of cancer had exceptionally low numbers of predicted coding SNV-drivers: this included neuroblastoma, thyroid cancer and renal cell carcinoma (RECA).

Low mean counts for SNV-drivers in coding regions has been argued by previous authors [57, 58], for example, by Martincorena *et al.* [59] in a recent study using a different argument based on the normalized ratio of non-synonymous to synonymous mutations (dN/dS). They also used a different dataset (we use ICGC data [52] in the discussion below, they used TCGA data [53]). Though with a similar variability by cancer type, an apparent point of difference is that Martincorena *et al.* argue for a lower mean count of only four SNV-drivers in coding regions, across the range of cancers they consider, which partly overlaps the *25* cancer types in our own survey. One reason for a difference can be alternative choices for the statistical significance level. However, a second difference originates more subtly from the classifier. To avoid bias, *CScape* was trained on balanced data by class, and hence would be expected to predict with approximately balanced false positives and false negatives. However, the number of true positives (SNV-drivers) is evidently very small in size, from the discussion above, and hence the classifier has a potential bias toward *overestimating* the number of positives: errors are accumulating at an equal rate for false positives and negatives but the positive class is actually very small in size. In consequence, a higher accuracy predictor is likely to predict *fewer* SNV disease-driver positives. Thus, these two estimation approaches do not necessarily disagree, but await more accurate prediction. *CScape* does have the major advantage over dN/dS of putatively identifying those SNVs which are driving cell proliferation.

The discussion just presented appears to ignore one issue: estimating driver counts by amalgamating data across different stages of disease could create a bias in a comparison across cancer types. The tumor mutational burden may increase with stage of disease and there could be unequal sampling rates by stage. Also, successful intervention may deplete the sampling size for later stages of disease, for some cancer types. Restricting to coding SNVs, our analysis [55], using *CScape*, indicated that the mean count increases with stage of disease for some types of cancer. Both early onset prostate cancer (typecode: EOPC) and prostate cancer (PRAD) have low mean counts for coding SNV-drivers which increase steadily with stage: *2.5* (stage I), *3.8* (IIA), *6.5* (IIIB) for EOPC and *4.4* (IIB), *7.6* (IIC), *16.4* (IIIB), *21.8* (IVA) for PRAD, respectively. However, prostate cancer develops slowly over an extended period which may allow for an accumulation of additional SNV-drivers. Overall, though, our analysis suggested that increasing numbers of SNV-drivers with stage of disease is an exception as a phenomenon, not the rule. More typical are RECA, which has a mean SNV-driver count which is steady and low at *3.4* (I), *3.3* (II), *3.2* (III) and *3.0* (IV). Similarly, esophageal cancer (ESCA) has a steady but higher count at *13.5* (I), *9.9* (II), *10.0* (III) and *12.2* (IVA). Using a comparison of stage I versus stage IV cancer, with a different argument and dataset, the same conclusion is reached by Martincorena *et al.* (Figure S4C [59]) who argue there is little evidence for significant increases in the number of SNV-drivers as disease progresses, across the cancer types they consider. Two caveats to our own analysis [55] are that we only consider samples labeled as primaries (from the ICGC dataset [52]), and that rare drivers (e.g. a recurrence of *r = 1* across the dataset) may accumulate with stage, while being subliminal for the classifier to identify.

In terms of establishing biological validity, a further question is the identification of those genes having embedded variants which are predicted disease-drivers. Since our focus is on sequence variants as drivers, in the discussion below we will label a gene as a potential driver if it has *at least one high confidence embedded SNV-driver*, that is, the SNV is labeled as a positive (disease-driver) with an FDR of 5%. We emphasize this is *only one definition of a driver-gene*. For predicting whether a gene is a driver, we could instead use data taken across the whole gene, rather than consider just embedded SNVs. This means we can use more data and feature groups, potentially improving test accuracy performance. For example, we could use the full set of mutations across the gene encoded via a normalized entropy measure, or full sequence alignments between mutated and reference unmutated gene. Both these two feature groups are used by *DeepDriver* [46], for example, based on use of a convolutional neural network. A variety of machine learning methods, such as convolutional neural networks [23, 60], and a variety of feature groups have been used for driver gene prediction. Features include high intra-gene mutation frequencies relative to the background rate, as used by *OncodriveCLUST* [61], for example, or feature groups such as ontology [62] or mutual exclusivity [63].

**Figure 2:**
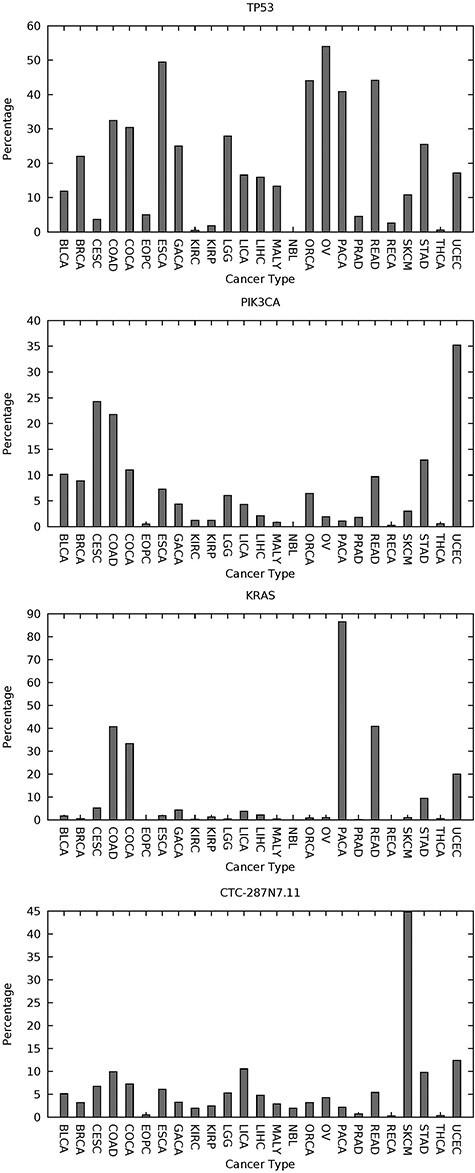
Four well-known cancer genes which can be labeled *common* drivers due to a higher incidence of predicted embedded *SNV*-drivers and an influence across multiple cancers. The *y*-axis gives the percentage incidence of at least one predicted high-confidence embedded *SNV*-driver (from use of *CScape* and using an FDR of 5%). The *x*-axis gives the typecodes of the 25 cancer types considered. These typecodes are matched with cancer name in supplementary table 1 of [55]. The figures for *TP53* and *KRAS* are reproduced from [55] under the Creative Commons License [64].

**Figure 3:**
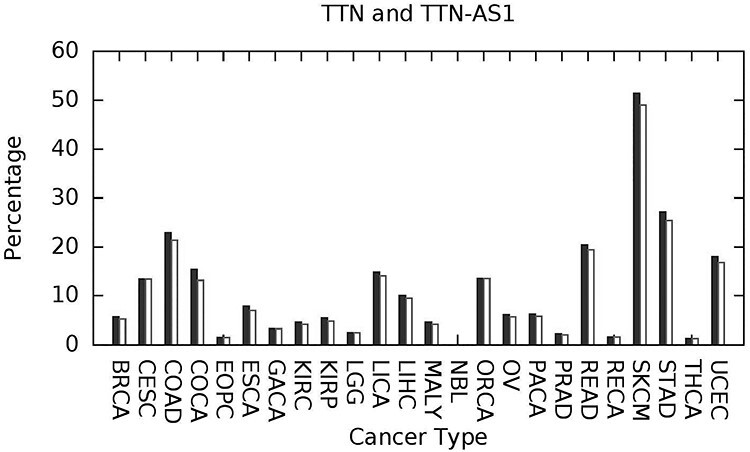
The long noncoding RNA gene *TTN-AS1* (white filled peaks) appears with a frequency, equal to, or slightly lower, than that for *TTN* (black filled peaks), in terms of percentage incidence of predicted embedded SNV-drivers (at an FDR of 5%). *TTN-AS1* is transcribed from the antisense strand of *TTN*, with the latter expressing the complex muscle protein *Titin*.

Using the criterion just stated only (*at least one embedded high confidence predicted SNV-driver*), we find that the spectrum of such predicted driver-genes is very individualized to the given tumor and it is drawn from small numbers of relatively more common driver-genes and long tails of infrequent driver-genes. As one would expect, *TP53* and *PIK3CA* are examples of genes with an influence across multiple cancer types and thus examples of relatively more common driver-genes. *KRAS* and the long noncoding RNA gene *CTC-297 N7.11* have a similar impact across multiple cancer types. These four genes are plotted in Figure 2, depicting the percentage of samples having at least one predicted high confidence SNV-driver. A less expected result was predicted widespread influence of the long noncoding RNA gene *TTN-AS1* (Figure 3). *TTN-AS1* is transcribed from the opposite strand to the gene *TTN* which has the largest number of exons of any gene in the human genome, and the longest single exon. The size of *TTN* would predispose toward random accumulation of false positives. However, *TTN-AS1* has also recently been proposed as an oncogene across various cancers [65] and one suggested mode of action has been dysregulation caused by the creation of competing endogenous RNA.

**Figure 4:**
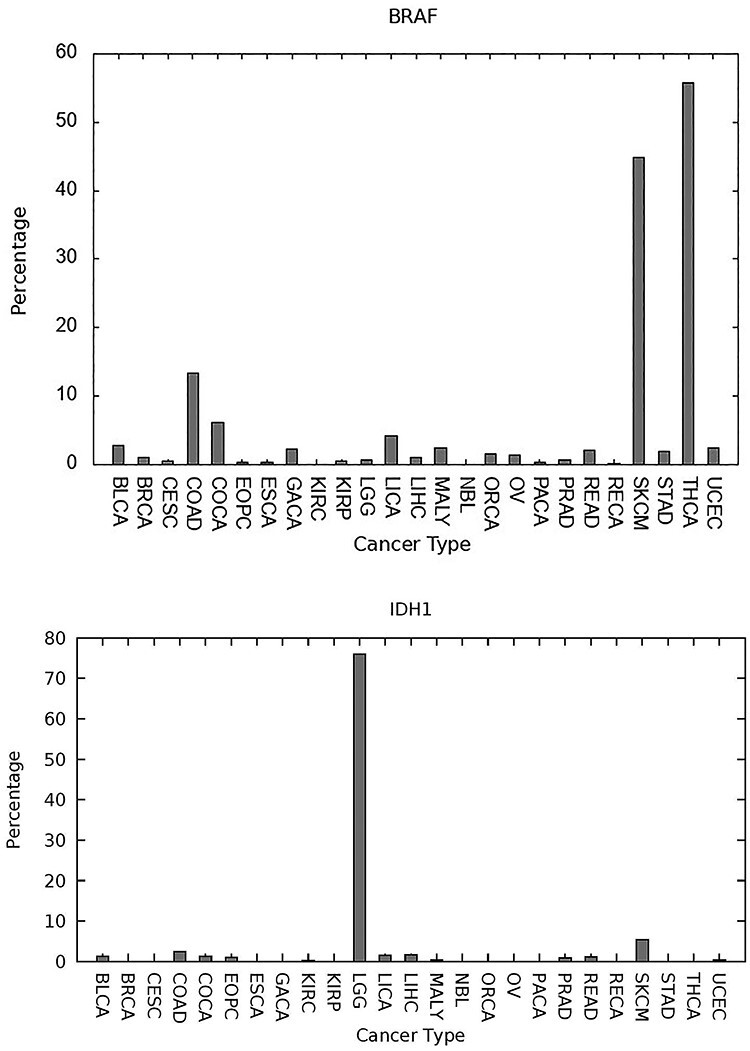
Two genes which have a more selective influence in certain contexts. *BRAF* (top) has at least one high-confidence embedded *SNV*-driver at significant percentage incidence levels for thyroid cancer (typecode:THCA), skin cutaneous melanoma (SKCM) and colon adenocarcinoma (COAD). *IDH1* (bottom) has a significant influence with LGG. Both genes are well documented within the cancer literature.

*BRAF* and *IDH1*, plotted in Figure 4, are predicted as having a more specific influence by cancer type, with a high percentage incidence of predicted SNV-drivers embedded in *BRAF* for skin cutaneous melanoma (44.8% of cases (typecode:SKCM)), thyroid cancer (55.8%, THY) and colon adenocarcinoma (13.4%, COAD). The relevance of *BRAF* mutations to all three of these cancers is extensively documented in the cancer research literature, see e.g. [66–68]. In Figure 4, we also depict *IDH1* with a significant percentage of predicted SNV-drivers for lower grade glioma (LGG), which has been extensively documented in the literature within the context of gliomas [69, 70]. Our survey [55] was restricted to *25* cancer types available from the International Cancer Genome Consortium database [52] and *IDH1* mutations occur in a wider range of cancer types than depicted here, including oligodendro-gliomas, astrocytomas, secondary glioblastomas and acute myeloid leukemia [71]. These two plots are illustrative of a motivation for further developing the cancer-specific prediction tools we have been discussing in this paper. Currently two *BRAF* inhibitors are approved for clinical use, *vemurafenib* and *dabrafenib*, targeted at melanoma. Several *IDH1* inhibitors have entered clinical trials (*AG-120*, *IDH-305*, *FT-2102* and *BAY1436032*) or been approved (*ivosidenib*) [71]. More accurate prediction tools may enable drug repurposing toward rare occurrence driver-genes which are distinct from their usual therapeutic context.

In summary, for *CScape* at least, we have quoted accuracies of 72% (*CScape*) and *74%* (*Cscape-somatic*) on balanced 50:50 unseen test data in coding regions of the cancer genome, and for SNVs. Thus, this classifier can successfully generalize to an extent, though not near to the levels quoted for the generic predictors of Section 1, when applied to non-cancer diseases. Cancer is, of course, a more difficult prediction context, as we now discuss. However, there is, at least, a *suggestion* that higher test accuracies can be achieved since *CScape* does attain a prediction performance of about *90%* if restricted to high confidence prediction.

In terms of biological validity, the genes discussed and listed in Figures 2 to 4 are well studied within the cancer literature and the suggested view of the cancer genome therefore looks plausible. Even if *CScape* is only partially accurate, provided there is no prediction bias involved, large sample sizes will average out the effects of noise, and so estimation of the relative significance of a driver-gene would be robust.

*CScape* is a pan-cancer prediction tool, rather than cancer-type specific. However, we observe from Figures 2 to 4 that there is evidence for transfer learning across cancer types, and a classifier such as *CScape* will gain from the larger data size inherent in a pan-cancer study. Though one may expect future gains from cancer-type-specific predictors, we have not found this to date, for these reasons.

## Discussion

Though able to predict with reasonable test accuracy, and with biological plausibility, further development of these cancer-specific prediction tools will need to master various challenges. One obvious challenge will be attaining a higher test accuracy performance. We have largely focused on SNVs in coding regions, but prediction with SNVs in noncoding regions is generally more difficult. On the other hand, prediction with indels is usually easier. One reason for this is that there is more sequence to assess and therefore more useable information from the various feature groups used.

As another issue, simply predicting the label as disease-driver or neutral carries limited information. More informative would be a predicted annotation as to why the variant is a driver. For SNVs in coding regions, one informative set of feature groups will be the predicted impact of a variant on protein structure or function. Various annotation tools have been proposed (e.g. [72]) and could be used for this purpose. One basic annotation beyond disease-driver will be whether the variant promotes gain-of-function or loss-of-function: a variant can, of course, deliver both of these simultaneously.

In terms of improving prediction accuracy, further gains will be made through the identification of additional feature groups which may be informative for driver status, or by alterations to the methodology used. As already commented, even if such additional feature groups are only weakly successful, data integration methodologies from machine learning can utilize a wide panel of such weakly informative data sources to construct a more accurate overall predictor. Care will have to be taken that additional feature groups do not introduce *circularity errors* by carrying implicit information about the predicted label, unfairly and positively biasing the predictor.

The test accuracy for cancer-specific SNV-status prediction currently lags the test accuracy performance for non-cancer prediction. One reason for this difference may be that prediction with cancer is mostly combinatoric. A particular substitution at a given location in a gene may be labeled as disease-driver or neutral depending on coding SNVs elsewhere, noncoding SNVs elsewhere, indels and a range of other causative events. The given substitution may be observed in a tumor, where it is part of the *driver combination* and promotes cell proliferation, or it may be isolated and hence inactive in a healthy individual. This amounts to a label noise since, even if a given single mutation has occurred, the label of disease-driver or neutral is potentially dependent on alterations elsewhere. Rather than predictors estimating driver status at a particular location, based on context data, it may be necessary to develop predictors in which predictions elsewhere in the cancer genome are used to modify the confidence estimate of driver status at a given location. Potentially, the driver-combination could involve SNVs, indels or other drivers based on copy number variation, or methylation, or major structural rearrangements, for example. The suggestion is that more accurate predictors may require multitrack input data, potentially covering the full set of alterations which could contribute to the driver-combination.

In summary, our discussion of these cancer-specific predictions indicates that machine learning methods are very suited to prediction with cancer datasets, given the very large and varied types of data involved, and the results of these studies suggest they will be increasingly capable of offering new insights.

**Key Points**

- A variety of tools have been presented for predicting the pathogenic impact of variants in the human genome, with test accuracies up to 90% stated for predicting the impacts of coding single nucleotide variants driving non-cancer disease.
- Similar tools for predicting the driver status of variants in the human cancer genome are at an earlier stage of development, and currently less accurate.
- For a given sample, driver genes in which predicted driver variants are embedded are frequently within well-documented cancer genes, such as TP53, PIK3CA, KRAS, BRAF and IDH1, and also within long tails of relatively infrequent driver-genes.
- The further development of such cancer-specific predictors may improve test accuracy by taking into account factors specific to cancer and will need to present predicted functional annotations beyond disease-driver versus neutral.

## Notes

**Mark Rogers** is currently a bioinformatics consultant based in Fort Collins, Colorado. He was formerly a Senior Research Associate at the University of Bristol and remains a Visiting Fellow there.

**Tom Gaunt** runs an interdisciplinary research team in the MRC Integrative Epidemiology Unit, University of Bristol. He completed his PhD in Human Genetics at the University of Southampton.

**Colin Campbell** is a Reader at the University of Bristol with interests in machine learning and medical bioinformatics. He graduated from Imperial College London (BSc) and Kings College London (PhD).
